# A Large Finer-grained Affective Computing EEG Dataset

**DOI:** 10.1038/s41597-023-02650-w

**Published:** 2023-10-25

**Authors:** Jingjing Chen, Xiaobin Wang, Chen Huang, Xin Hu, Xinke Shen, Dan Zhang

**Affiliations:** 1 Dept. of Psychology, School of Social Sciences, Tsinghua University, Beijing, China; 2 Tsinghua Laboratory of Brain and Intelligence, Tsinghua University, Beijing, China; 3 Dept. of Psychiatry, School of Medicine, University of Pittsburgh, Pittsburgh, USA; 4 Dept. of Biomedical Engineering, School of Medicine, Tsinghua University, Beijing, China

**Keywords:** Emotion, Human behaviour

## Abstract

Affective computing based on electroencephalogram (EEG) has gained increasing attention for its objectivity in measuring emotional states. While positive emotions play a crucial role in various real-world applications, such as human-computer interactions, the state-of-the-art EEG datasets have primarily focused on negative emotions, with less consideration given to positive emotions. Meanwhile, these datasets usually have a relatively small sample size, limiting exploration of the important issue of cross-subject affective computing. The proposed Finer-grained Affective Computing EEG Dataset (FACED) aimed to address these issues by recording 32-channel EEG signals from 123 subjects. During the experiment, subjects watched 28 emotion-elicitation video clips covering nine emotion categories (amusement, inspiration, joy, tenderness; anger, fear, disgust, sadness, and neutral emotion), providing a fine-grained and balanced categorization on both the positive and negative sides of emotion. The validation results show that emotion categories can be effectively recognized based on EEG signals at both the intra-subject and the cross-subject levels. The FACED dataset is expected to contribute to developing EEG-based affective computing algorithms for real-world applications.

## Background & Summary

Affective computing, aimed at enabling intelligent systems to recognize, interpret, and respond to people’s affective states, has drawn enthusiasm in various fields, including artificial intelligence, human-computer interaction, education, etc.^1,2^. In recent years, electroencephalogram (EEG) has gained increasing attention in the field of affective computing^3^. Unlike behavioural modalities such as voice, facial expression, and gestures that might be consciously disguised or restrained, EEG can objectively measure emotional states by recording people’s brain signals directly^4^. Compared with other neuroimaging technologies, EEG devices offer advantages such as relatively low cost and high portability, making them promising candidates for practical affective computing applications^5^. However, while research has demonstrated the feasibility of affective state decoding based on EEG signals, efforts are still needed to bridge the research-to-practice gap for EEG-based affective computing techniques towards real-world applications^6^.

First, while the importance of accurate decoding of positive emotions is acknowledged for real-world affective computing applications^7^, existing EEG-based affective computing studies have mainly used classical emotion theories with an oversimplified categorization of positive emotions^6,8^. For example, among Ekman’s six basic emotions, only “happiness” can be considered positive^9^. Considering that people usually experience positive emotions more frequently than negative emotions in their daily lives^10^, the relatively limited categorization of positive emotions may fail to effectively describe one’s affective states during possible affective-computing application scenarios^7^. Psychologists have called for a more balanced view of both the negative and positive side of emotion^11,12^, and emerging neuroscience studies have provided preliminary support for the decoding of discrete positive emotions. For instance, inspired by recent positive emotion theories^12^, distinct neural representations of positive emotions, such as joy, amusement, tenderness, etc., have been revealed with a video-watching paradigm for emotion elicitation^8,13–15^. However, publicly available EEG datasets have not sufficiently emphasized the positive side of emotion. A finer-grained emotion categorization, preferably with a special focus on positive emotions, is needed for datasets that better fulfill the needs of real-world affective computing applications^6^.

Second, emotion recognition that can be “plug-and-play” is always preferred in practical scenarios due to its time-saving and good user experience^16^. However, individual differences in people’s emotional experiences and the correspondingly individualized emotion-related EEG activities have posed challenges to the development of algorithms for cross-subject affective computing^4^. Indeed, substantial drops in the performance from intra-subject to cross-subject emotion recognition have been consistently reported^17,18^, hindering seamless emotion recognition usage. Due to the time and labour cost for EEG data collection, available benchmark datasets usually have a relatively limited sample size (20~60 subjects)^19–22^. A dataset with a larger sample size, however, may help address the cross-subject affective computing challenges, as the extraction of subject-invariant representation of emotional states could benefit from an increase in the subject number^23^. In particular, the recent rise of deep learning methods has brought new possibilities for cross-subject challenges and also placed higher demands on the sample size^6,24^. With the development of data augmentation techniques^25^, the expected positive effects of the increased sample size could be amplified.

The present Finer-grained Affective Computing EEG Dataset (FACED) aims to address these issues by recording EEG signals from 123 subjects who watched 28 emotion-elicitation video clips covering nine emotion categories (amusement, inspiration, joy, tenderness; anger, fear, disgust, sadness, and neutral emotion). The sample size of over 100 subjects is expected to facilitate the cross-subject affective computing research. The EEG data were recorded using 32 electrodes according to the international 10–20 system. For each video clip, subjective ratings were obtained for all subjects, covering the dimensions of the four negative and four positive emotions, as well as arousal, valence, familiarity, and liking. For validation, we used a classical machine learning algorithm^26^ for both intra-subject and cross-subject affective computing and a state-of-the-art algorithm utilizing a contrastive learning framework^4^ for cross-subject affective computing. The features of the FACED dataset are summarized in Table 1. The validation supports the effectiveness of nine-category cross-subject affective recognition. The dataset is open-access for research purposes: 10.7303/syn50614194.

**Table 1:** The summary of key features of the FACED dataset.

Key features of the FACED dataset		
Number of subjects	123	
Emotion category	9	anger, fear, disgust, sadness, amusement, inspiration, joy, tenderness, and neutral emotion
Number of video clips	28	three clips for each negative/positive emotion and four clips for the neutral emotion
Self-reporting ratings (continuous scale of 0–7)	12 items	anger, fear, disgust, sadness, amusement, inspiration, joy, and tenderness valence, arousal, liking, and familiarity
Recorded signals	32-channel EEG	

## Methods

### Stimuli and experiment procedure

Twenty-eight video clips were used to elicit nine categories of emotion (four negative emotions: anger, disgust, fear, and sadness; four positive emotions: amusement, inspiration, joy, and tenderness; and the neutral emotion). The selection of emotion labels is based on the following considerations. The four negative emotions were derived from Ekman’s six basic emotions^9^, while the selection of the four positive emotions was based on the latest advancements in psychology and neuroscience, as well as specific application requirements: Recent neuroscience studies have identified three positive emotions (inspiration, joy, and tenderness) as being representative^8,13^, and amusement is frequently encountered in application scenarios like human-computer interactions^27,28^. The emotion-evoking video clips were selected from various databases, including the FlimStim database^29^, the database of positive emotional videos^8,13^, the standardized database of Chinese emotional videos^30^, and the database of emotion profile videos^31^. Each negative/positive emotion category had three video clips, while the neutral emotion category had four clips. On average, these video clips lasted about 66 seconds, with duration ranging from 34 to 129 seconds. The details of each video clip are provided in Supplementary Table S1.

**Fig. 1:**
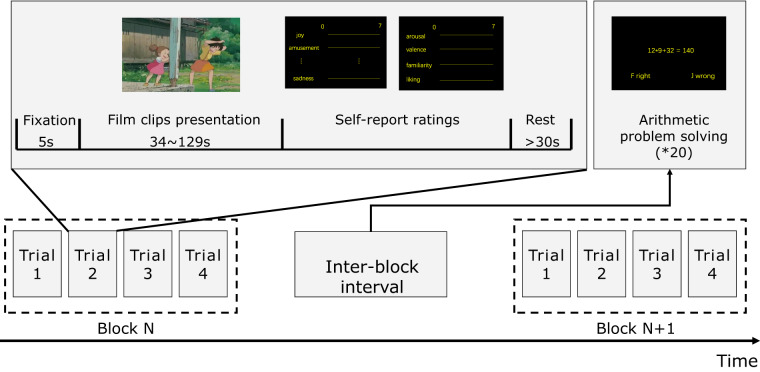
Experimental procedure.

Figure 1 demonstrates the experimental procedure. During the experiment, subjects were seated approximately 60 cm away from a 22-inch LCD monitor (Dell, USA). Each trial began with subjects focusing on a fixation cross for 5 seconds, followed by watching a video clip. The sound of video clips was played through stereo speakers (Dell, USA). After each video clip, subjects were required to report their subjective experiences during the video-watching on 12 items, including anger, fear, disgust, sadness, amusement, inspiration, joy, and tenderness, as well as valence, arousal, liking and familiarity. Subjects provided ratings on a continuous scale of 0–7^31,32^ for each item and then had at least 30 seconds of rest before starting the subsequent trial. Here, for the valence item, 0 indicated “very negative” and 7 indicated “very positive”. For the other items, 0 indicated “not at all” and 7 indicated “very much”. The meaning of the 12 items was explained to the subjects before the experiment.

To minimize the possible influence of alternating valence, video clips with the same valence (e.g., positive) were presented successively as a block of four trials. Consequently, there were three positive blocks, three negative blocks, and one neutral block. Between two blocks, subjects completed 20 arithmetic problems to minimize the influence of previous emotional states on the subsequent block^33^. When answering the arithmetic problems, if subjects did not complete a problem in 4 seconds, it would be skipped, and the next problem would be presented. The order of the video clips within each block and the seven blocks was randomized across subjects. Before the experiment, subjects performed one practice trial to become familiar with the procedure. The experimental procedure was programmed with Psychophysics Toolbox 3.0 extensions^34^ in MATLAB (The Mathworks, USA).

### Subjects

A total of 123 subjects (75 females, mean age = 23.2 years, ranging from 17 to 38 years; all native Chinese) were recruited for the experiment. None of the subjects have reported a history of neurological or psychiatric disorders. The subjects were de-identified and indexed as S000~S122. The study was approved by the local Ethics Committee of Tsinghua University (THU201906), and informed consent was obtained from all subjects.

### Data acquisition

The EEG signals were recorded using a wireless EEG system (NeuSen.W32, Neuracle, China) at a sampling rate of 250 or 1000 Hz. Thirty-two wet electrodes (Ag/AgCl electrodes with conductive gel) were placed according to the international 10–20 system. The impedance was kept below 10 kOhm throughout the experiment. Our experiment was conducted in two cohorts in two distinct time periods, involving two non-overlapping groups of subjects. Cohort 1 included participants sub000 to sub060, while cohort 2 encompassed participants sub061 to sub122. The data in both cohorts was collected with the same experimental procedure. In the experiment, we initially used a sampling rate of 250 Hz, which is comparable with other datasets^19,35^. However, the sampling rate was switched to 1000 Hz^18^ later to provide richer information for data analysis. The sampling rate for each subject during recording is provided in Recording_info.csv, along with the dataset. Data in both cohorts were recorded with the reference electrode at CPz and the ground electrode at AFz. The reference and ground electrode were defaulted by the EEG amplifier, which was also used in other emotion-related studies^31,36^. Note that the 32-electrode coverage available in the present dataset allowed multiple re-referencing methods (e.g., the common average or the average of both mastoids) by simple linear computations in subsequent analysis. The spatial placement of the electrodes in the two cohorts is the same, although 6 of them have different names due to the device setting. The electrode information for both cohorts can be found in Supplementary Tables S2, S3. During the experiment, the recorded EEG signals were synchronized to the experimental procedure by sending triggers to the EEG recording system with a serial port when events occurred, which is a common practice in data-task synchronization in EEG-based experiments^37^. The event information during the experiment is listed in Table 2.

**Table 2:** The event information during the experiment.

Event	Trigger value	
Experiment start	100	Successive triggers of 100 as the start of the experiment
Video index	1–28	The index of each video clip
Video clip start	101	The start of each video clip
Video clip end	102	The end of each video clip

### Data pre-processing

The dataset was collected in a regular office environment resembling possible practical application scenarios^19,38^. Then, to validate the dataset, we conducted a pre-processing procedure to enable further analysis. The pre-processing process was conducted based on the MNE toolbox^39^, version 1.2.1, with Python 3.10. Codes for data pre-processing were provided together with the dataset. First of all, the unit for the recorded EEG signal was adjusted to μV. Then, the last 30 seconds of each video clip were selected to capture the maximal emotional responses^4,8^ based on the timing of events that indicates the end of each video clip (i.e., “Video clip end” in Table 2). Then, the sampling rates of EEG were adjusted to 250 Hz (downsampled when necessary). Next, filtering, interpolation, and independent component analysis (ICA) were conducted to remove possible motion and ocular artifacts, which was similar to the pre-processing pipelines of other datasets like DEAP and SEED. Specifically, a bandpass filter from 0.05 to 47 Hz was applied to the EEG signals with the MNE *filter()* functions. Following that, samples whose absolute values exceeded three times the median absolute value in each 30-second trial were defined as outliers^4^. In each 30-second EEG trial, if the proportion of outliers for an electrode exceeded 30%, this electrode was defined as a bad electrode and was interpolated, following previous studies^4,40^ with the MNE *interpolate_bads()* function. Then, the ICA method was performed. The independent components (ICs) containing ocular artifacts were automatically defined and rejected by using FP1/2 as the proxy for electro-oculogram with the MNE *ica.find_bads_eog()* and *ica.exclude* functions. Next, the cleaned EEG signals were re-referenced to the common average reference. Finally, the order of electrodes in cohort 1 was adjusted to be consistent with cohort 2. Note that all the data pre-processing was conducted offline. The raw EEG data are provided and hereby available to the users. We also provide the pre-processed data to promote more efficient use of the present dataset. We recommend that users read the released pre-processing code before using the pre-processed data to develop a more detailed grasp of the implementation. Nevertheless, users can design their own pre-processing pipeline and apply it to the raw data according to their specific needs (e.g., considering sufficient artifact removal towards electromyography, electrocardiogram, and channel noise).

We also provide commonly-used EEG features, including differential entropy (DE)^41^ and power spectral density (PSD)^42^ for our dataset. The DE and PSD features were obtained from the pre-processed data within each non-overlapping second at 5 frequency bands (delta band: 1–4 Hz, theta band: 4–8 Hz, alpha band: 8–14 Hz, beta band: 14–30 Hz and gamma band: 30–47 Hz). The formula to calculate DE and PSD followed the practice in the SEED dataset (https://bcmi.sjtu.edu.cn/home/seed/seed-iv.html).

1 $$PSD=E[{x}^{2}]$$

2 $$DE=\frac{1}{2}ln\left(2\pi e{\sigma }^{2}\right)$$

Where *x* is the EEG signal, *σ* is the variance of the EEG signal.

## Data Records

The FACED dataset is available in Synapse^43^ and stored in the “FACED” repository (Project SynID: syn50614194) at the website 10.7303/syn50614194. As shown in Table 3, the current dataset contains data records from 123 subjects. For each subject, we provide raw EEG data and event data in the “.bdf” file format, self-reported ratings in the MATLAB “.mat” format, pre-processed EEG data in the Python “.pkl” format, DE and PSD features in the Python “.pkl” format. The pre-processed data were obtained after applying the pre-processing pipeline described in the Methods section to the raw EEG data. For each subject, the pre-processed EEG data is presented as a 3-dimensional matrix of VideoNum*ElecNum*(TrialDur*SampRate). The number of video clips is 28. The order of video clips in the pre-processed data was reorganized according to the index of video clips, as reported in Supplementary Table S1. The number of electrodes is 32. The order of electrodes is provided in Supplementary Table S3. The duration of each EEG trial is 30 seconds, and the sampling rate of pre-processed EEG data is 250 Hz. For each subject, the DE and PSD feature is a 4-dimensional matrix of VideoNum*ElecNum*TrialDur*FreqBand. There are 5 frequency bands, corresponding to delta, theta, alpha, beta, and gamma band, respectively.

**Table 3:** Data records in the FACED dataset.

File name	Content
Dataset_description.md	Description of the dataset
Task_event.xslx	Event information during the experiment
Electrode_Location.xslx	Electrode information
Stimuli_info.xslx	Details of the video clips
Recording_info.csv	Age, gender, sampling rate, and the units of EEG signal for each subject
DataStructureOfBehaviouralData.xslx	Data structure of the behavioural data
Data/subXXX/data.bdf, evt.bdf	Raw EEG and event data
Data/subXXX/After_remarks.mat	Self-reporting ratings and the performances of the inter-block arithmetic task
Processed_Data/subXXX.pkl	Pre-processed EEG data 3-dimensional matrix of VideoNum*ElecNum*(TrialDur *SampRate)
EEG_Features/DE/ subXXX.pkl	DE feature 4-dimensional matrix of VideoNum*ElecNum*TrialDur*FreqBand
EEG_Features/PSD/ subXXX.pkl	PSD feature 4-dimensional matrix of VideoNum*ElecNum*TrialDur*FreqBand
Code	Codes for data pre-processing and validation
README.md	Usage notes for data and codes

*Note*: The subXXX indicates sub000~sub122.

The data structure of behavioural data is shown below in Table 4. For each subject, the behavioural data includes self-report ratings on 12 items for each video. Additionally, task performances, including accuracy and response time, for the arithmetic problem-solving task during each inter-block interval are also provided. The unit for the response time is in seconds.

**Table 4:** The data structure of the behavioural data.

Field		
score	Rating scores of 12 items for the 28 video clips	0–7 Item order: “joy”,“tenderness”,“inspiration”,“amusement”,“anger”,“disgust”,“fear”,“sadness”,“arousal”,“valence”,“familiarity”, “liking”
trial	Presentation orders of the 28 video clips	1–28
vid	Video indexes of the 28 video clips	1–28
Accuracy	Averaged accuracy of the arithmetic task during each inter-block interval	Ranging from 0 to 1
ResponseTime	Response times of the 20 arithmetic problems during each inter-block interval	The unit for response time is in seconds

## Technical Validation

### Behavioural data validation

**Fig. 2:**
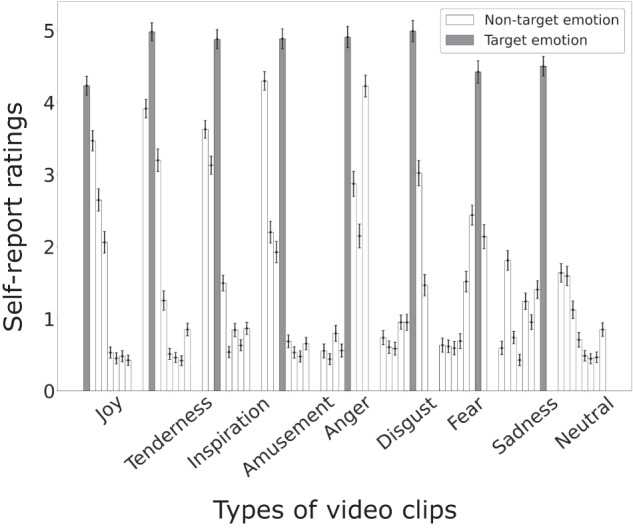
The subjects’ ratings on the emotional experience items. For each category of video clips, the eight bars indicate mean ratings of the video clips on joy, tenderness, inspiration, amusement, anger, disgust, fear, and sadness (from left to right). The gray bars indicate the ratings on the corresponding target emotion; The white bars indicate the ratings on the non-target emotion. The error bars indicate standard errors.

To assess the effectiveness of the video-watching paradigm in eliciting the targeted emotions, we conducted repeated measures analyses of variance (rmANOVA) and post-hoc tests on the subjects’ emotional ratings. As illustrated in Fig. 2, the category of video clips that was expected to elicit one specific emotion indeed yielded the highest self-report ratings for the target emotion (rmANOVA *p* < 0.001 for all emotion items and post-hoc tests showed significantly higher ratings for the target emotion, *p* < 0.05, false discovery rate corrected). At the same time, neutral video clips received low arousal ratings and moderate valence ratings (average arousal score: 1.41; average valence score: 3.18). The results validate the efficacy of the current video-watching paradigm. The self-report ratings on arousal, valence, familiarity, and liking are demonstrated in Supplementary Fig. S1. The self-report ratings for each video clip on all 12 items are provided in Supplementary Table S4.

### EEG data validation

**Fig. 3:**
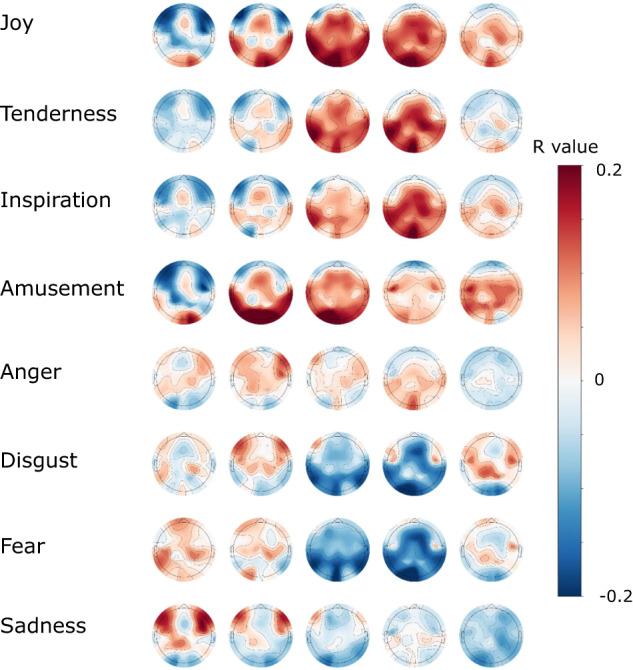
The topographies of the correlation coefficients between the relative spectral powers at the five frequency bands and the subjects’ ratings on the eight emotion items.

Pearson’s correlations between the relative EEG spectral powers and self-reported emotional ratings were computed to identify the neural correlates of emotional experiences. The relative spectral powers were defined as the ratio between the sum of the spectral powers in the frequency band of interest (delta, theta, alpha, beta and gamma band) and the sum of the full-band spectral power. Specifically, for every 30-second EEG trial of each video, the trial was divided into 1-second epochs. Then, Fourier Transform was conducted for each 1-second epoch, and the relative spectral powers for each video clip were calculated by obtaining the median of all 30 1-second epochs. Then, the correlation was calculated between each subject’s ratings on a specific emotion item for 28 video clips and her/his relative spectral powers for the same 28 video clips at each electrode. The topographies of the averaged Pearson’s correlation coefficients over all subjects were demonstrated in Fig. 3 and Supplementary Fig. S2. Distinct EEG correlates of different emotional experiences were observed spatially and spectrally, showing comparable magnitudes of correlation coefficients with one previous study^8^. These results suggest that the video-elicited EEG signals contain emotion-related information, providing the neural basis for EEG-based emotion recognition.

### Classification analysis

**Fig. 4:**
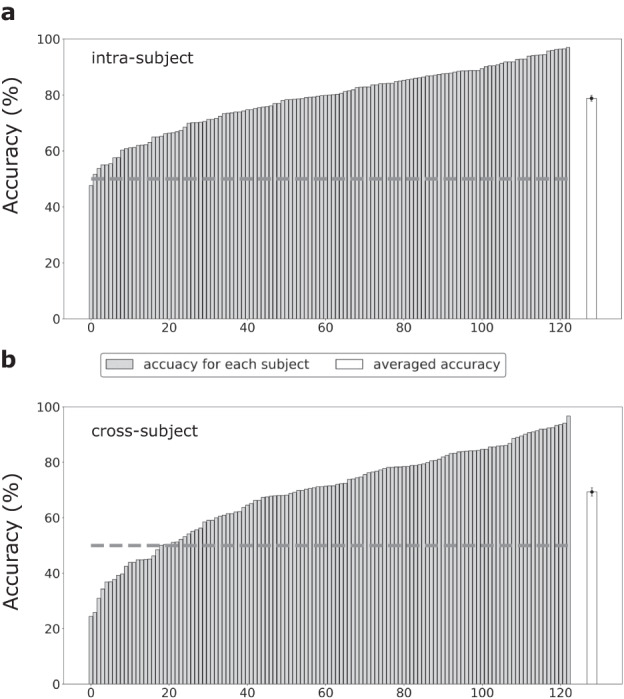
The classification accuracies for each subject with DE + SVM in the binary classification of (**a**) intra-subject and (**b**) cross-subject emotion recognition. The subjects are re-ranked according to their classification accuracies, increasing from left to right. The light gray bars indicate accuracies for each subject, and the white bars indicate averaged accuracies across all subjects. The error bars of white bars indicate the standard error across all subjects. The dotted gray line indicates the chance level of binary classification.

Classification analysis was further conducted to validate the utility of the data records in two parts: 1. Binary classification of positive and negative emotional states was performed to directly compare with previous studies. 2. Classification of the nine-class emotional states was conducted to test whether the present dataset could support a finer-grained emotion recognition. The classification of emotional states was conducted on a 1-second time scale. In the first part, the classical method based on DE features and support vector machine (SVM)^26^ was used for intra-subject and cross-subject emotion recognition. Here, anger, disgust, fear, and sadness were labelled negative, while joy, amusement, inspiration, and tenderness were labelled positive. The neutral emotion was excluded due to an imbalanced data amount (4 neutral video clips vs. 12 positive/negative video clips). The recognition was carried out using the pre-processed data with a ten-fold procedure. In the intra-subject emotion recognition, for all positive/negative video clips, 90% of EEG data in each video clip served as the training sets, and the remaining 10% was used as the testing sets for each subject. In the cross-subject emotion recognition, the subjects were divided into 10 folds (12 subjects for the first nine folds, and 15 for the 10^th^ fold). Then, nine-fold subjects were used as the training sets, and the remaining subjects were used as the testing sets. The procedure was repeated 10 times and the classification performances were obtained by averaging accuracies for 10 folds. The classification accuracies of 78.8 ± 1.0% and 69.3 ± 1.5% (mean ± standard error, the same below for the reported classification accuracies) were obtained for the intra-subject and cross-subject emotion recognition, respectively. Both the intra-subject and cross-subject performances were comparable with previous studies using the same classification methods^26,44^. A drop in performance was also observed in the cross-subject recognition compared with the intra-subject recognition, consistent with findings from previous studies^17,18^. The classification accuracies for each subject were demonstrated in Fig. 4. Both intra-subject and cross-subject classification reveal substantial individual differences, underscoring the value of large-scale datasets in better characterizing population attributes.

**Fig. 5:**
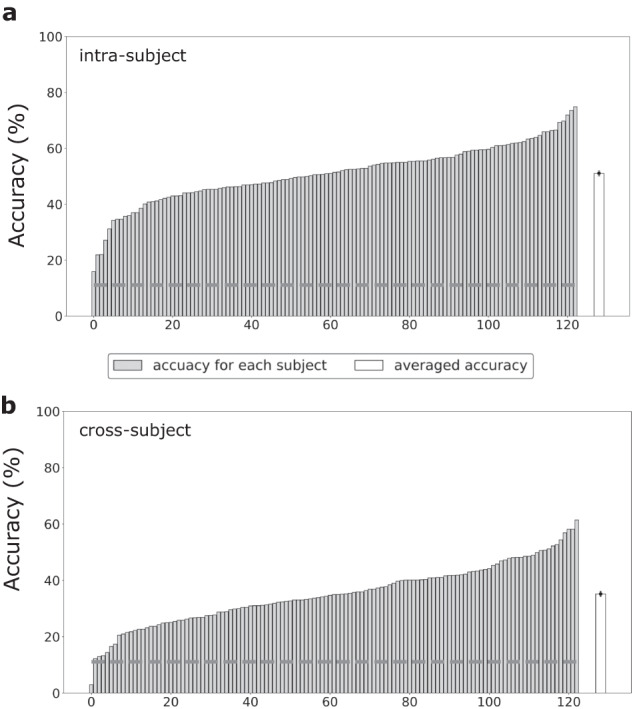
The classification accuracies for each subject with DE + SVM in the nine-category classification of (**a**) intra-subject and (**b**) cross-subject emotion recognition. The subjects are re-ranked according to their classification accuracies, increasing from left to right. The light gray bars indicate accuracies for each subject, and the white bars indicate averaged accuracies across all subjects. The error bars of white bars indicate the standard error across all subjects. The dotted gray line indicates the chance level of nine-category classification.

In the second part, we performed a classification of the nine-class emotional states to assess if the present dataset could support more fine-grained emotion recognition. The same classification procedure (DE + SVM with a 10-fold cross-validation, as detailed above) was conducted to classify joy, tenderness, inspiration, amusement, anger, disgust, fear, sadness, and neutral emotions. The achieved accuracies were well above the chance level (intra-subject: 51.1 ± 0.9%; cross-subject: 35.2 ± 1.0%), indicating the feasibility of decoding multiple emotional states based on EEG signals. The classification accuracies for each subject were demonstrated in Fig. 5.

**Fig. 6:**
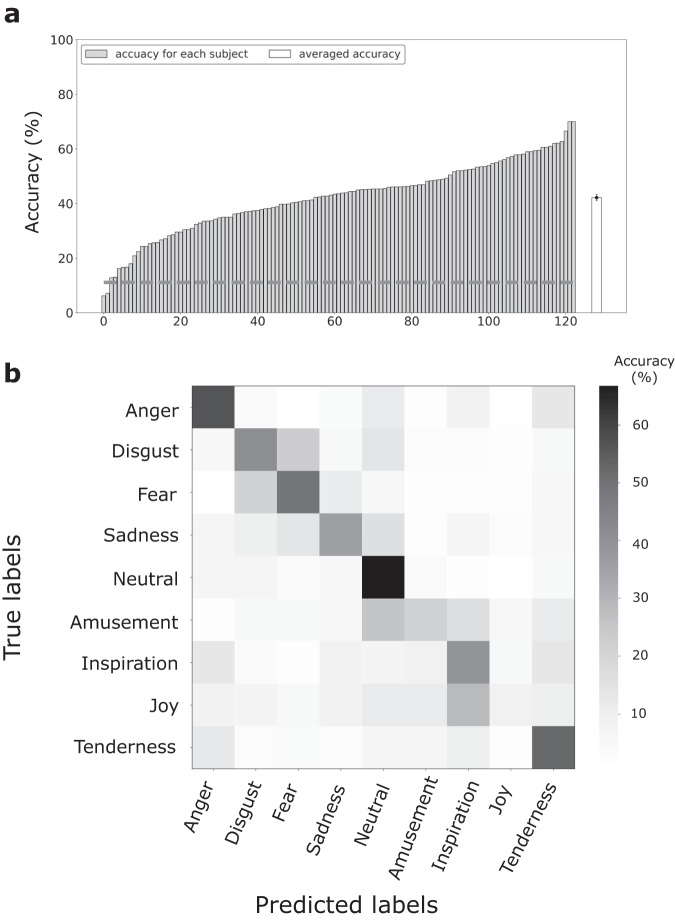
(**a**) The classification accuracies for each subject with the CLISA algorithm for the nine-category cross-subject emotion recognition and (**b**) the averaged confusion matrix. The subjects are re-ranked according to their classification accuracies, increasing from left to right. The light gray bars indicate accuracies for each subject, and the white bar indicate averaged accuracies across all subjects. The error bar of the white bar indicates the standard error across all subjects. The dotted gray line indicates the chance level of the nine-category classification.

Moreover, we also employed one state-of-the-art algorithm named Contrastive Learning for Inter-Subject Alignment (CLISA)^4^ for the cross-subject recognition of the nine emotion categories. The objective of CLISA was to reduce inter-subject differences by maximizing the similarity in EEG signal representations among subjects when exposed to the same emotional stimuli, as opposed to different ones. Subsequently, the inter-subject-aligned EEG representations were used to extract features for emotion classification, which are expected to be relatively stable across subjects. Due to its state-of-the-art cross-subject emotion recognition performance on several EEG datasets, we selected the CLISA algorithm to validate the newly proposed FACED dataset. A classification accuracy of 42.4 ± 1.2% was achieved with a ten-fold procedure, demonstrating a 7.2% improvement in the nine-emotion classification cross-subject performance. The accuracy was also comparable with one previous study^4^. The classification accuracies based on CLISA are shown in Fig. 6. The classification results supported the potential to boost the cross-subject performance by integrating the latest advancements in deep learning.

In summary, the technical validation of self-reporting ratings indicated that the current video-watching paradigm effectively elicited the targeted emotions. The correlation analysis between self-reporting ratings and EEG signals indicated that the video-elicited EEG signals contained emotion-related information. The classification analysis further showed that the emotion categories could be successfully recognized based on the EEG signals at both the intra-subject and the cross-subject level. These technical validation results collectively support the validity and reliability of the present dataset.

### Supplementary information

Supplementary Information

## Data Availability

All the codes used for the data pre-processing and the technical validation are publicly available together with the FACED datasets in Synapse (10.7303/syn50614194). The codes were developed in Python 3.10. These codes can be executed on Linux and Windows. All required packages are listed in the torch_ubuntu.yml and torch_win.yml files. The README file under the Code file provides a detailed explanation of the procedure to reproduce the validation results using the codes and data.
