# Supplementary material for: A Large Finer-grained Affective Computing EEG Dataset
Source: Sci Data. 2023 Oct 25;10:740. doi: 10.1038/s41597-023-02650-w (PMC10600242; doi:10.1038/s41597-023-02650-w)
Supplement: Supplementary file 1 — Supplementary Information [file 41597_2023_2650_MOESM1_ESM.docx]

Supplementary Information

Fig. S1 The subjects’ ratings on the dimensional emotional experience items (arousal, valence, familiarity, and liking). For each item, diagonal lines (downward to right) indicate the positive video clips (joy, tenderness, inspiration, amusement, from left to right), diagonal lines (upward to right) indicate the negative video clips (anger, disgust, fear, sadness, from left to right), and horizontal lines indicate neural video clips. The error bar indicated standard errors.


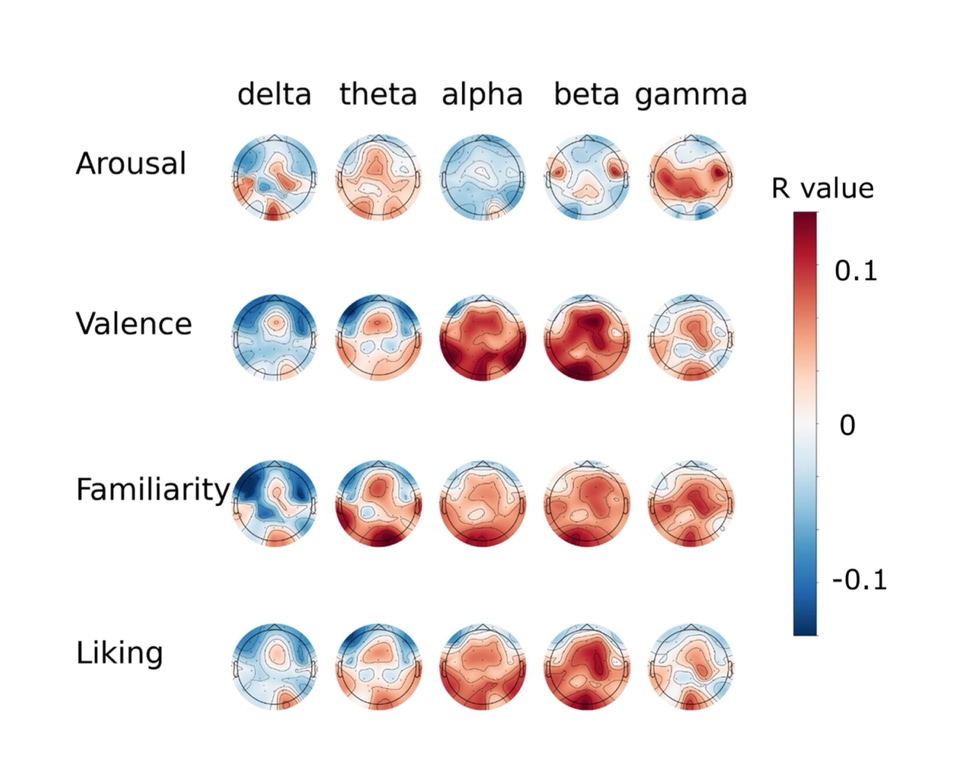


Fig. S2 The topographies of the correlation coefficients between the relative spectral powers and subjects’ ratings on arousal, valence, familiarity, and liking.

**Table S1. Information about the video stimuli**

| **Video index** | **Duration（s）** | **Source Film** | **Source Database** | **Targeted**  **Valence** | **Targeted Emotion** |
| --- | --- | --- | --- | --- | --- |
| 1 | 81 | The Tokyo Trial | DCEF | Negative | Anger |
| 2 | 64 | Documentary about the Nanjing Massacre | THU-EP | Negative | Anger |
| 3 | 73 | City of Life and Death | DCEF | Negative | Anger |
| 4 | 79 | Trainspotting | FilmStim | Negative | Disgust |
| 5 | 69 | Indiana Jones and the Last Crusade | FilmStim | Negative | Disgust |
| 6 | 91 | Hellraiser | FilmStim | Negative | Disgust |
| 7 | 56 | The Shining | FilmStim | Negative | Fear |
| 8 | 60 | The Shining | \ | Negative | Fear |
| 9 | 106 | The Exorcist | FilmStim | Negative | Fear |
| 10 | 45 | In Bruges | PED | Negative | Sadness |
| 11 | 60 | Departures | PED | Negative | Sadness |
| 12 | 82 | Gangs of New York | PED | Negative | Sadness |
| 13 | 35 | Blue | FilmStim | Neutral | / |
| 14 | 44 | Blue | FilmStim | Neutral | / |
| 15 | 39 | Blue | FilmStim | Neutral | / |
| 16 | 43 | Blue | FilmStim | Neutral | / |
| 17 | 56 | Modern Times | PED | Positive | Amusement |
| 18 | 70 | Minions | PED | Positive | Amusement |
| 19 | 73 | Mr. Bean | PED | Positive | Amusement |
| 20 | 129 | Forrest Gump | PED | Positive | Inspiration |
| 21 | 77 | The Theory of Everything | PED | Positive | Inspiration |
| 22 | 76 | The Shawshank Redemption | PED | Positive | Inspiration |
| 23 | 34 | My Neighbor Totoro | PED | Positive | Joy |
| 24 | 37 | Night at the Museum Ⅲ | PED | Positive | Joy |
| 25 | 68 | Harry Potter Ⅰ | PED | Positive | Joy |
| 26 | 63 | The Pursuit of Happiness | PED | Positive | Tenderness |
| 27 | 54 | Juno | PED | Positive | Tenderness |
| 28 | 77 | Sex and the City Ⅱ | PED | Positive | Tenderness |

Notes: DCEF refers to the standardised database of Chinese emotional film clips (Ge et al., 2019), THU-EP refers to the emotion profile database (Hu et al., 2022), PED refers to the positive emotion database (Hu et al., 2017; Hu et al., 2019), and FlimStim refers to the database created by Schaefer (2010). Please find more detailed information in the corresponding papers.

**Table S2. Electrode information for the first cohort (sub000 ~ sub060)**

| 1 | Fp1 | 9 | FC2 | 17 | A1 | 25 | P4 |
| --- | --- | --- | --- | --- | --- | --- | --- |
| 2 | Fp2 | 10 | FC5 | 18 | A2 | 26 | T5 |
| 3 | Fz | 11 | FC6 | 19 | CP1 | 27 | T6 |
| 4 | F3 | 12 | Cz | 20 | CP2 | 28 | PO3 |
| 5 | F4 | 13 | C3 | 21 | CP5 | 29 | PO4 |
| 6 | F7 | 14 | C4 | 22 | CP6 | 30 | Oz |
| 7 | F8 | 15 | T3 | 23 | Pz | 31 | O1 |
| 8 | FC1 | 16 | T4 | 24 | P3 | 32 | O2 |

**Table S3. Electrode information for the second cohort (sub061~sub122)**

| 1 | FP1 | 9 | FC2 | 17 | CP1 | 25 | P8 |
| --- | --- | --- | --- | --- | --- | --- | --- |
| 2 | FP2 | 10 | FC5 | 18 | CP2 | 26 | PO3 |
| 3 | Fz | 11 | FC6 | 19 | CP5 | 27 | PO4 |
| 4 | F3 | 12 | Cz | 20 | CP6 | 28 | Oz |
| 5 | F4 | 13 | C3 | 21 | Pz | 29 | O1 |
| 6 | F7 | 14 | C4 | 22 | P3 | 30 | O2 |
| 7 | F8 | 15 | T7 | 23 | P4 | 31 | HEOR |
| 8 | FC1 | 16 | T8 | 24 | P7 | 32 | HEOL |

Note: Electrodes in the first cohort include Fp1/2, Fz, F3/4, F7/8, FC1/2, FC5/6, Cz, C3/4, T3/4, CP1/2, CP5/6, T5/6, Pz, P3/4, PO3/4, Oz, O1/2, A1/2 (left and right mastoids), while electrodes in the second cohort include Fp1/2, Fz, F3/4, F7/8, FC1/2, FC5/6, Cz, C3/4, T7/8, CP1/2, CP5/6, Pz, P3/4, P7/8, PO3/4, Oz, O1/2, HEOL/HEOR. The spatial placement of the electrodes in two cohorts are all the same, although 6 of them have different names due to the device setting. Here, T3/4 channels is corresponding to T7/8 channels; T5/6 is corresponding to P7/8; A1/A2 is corresponding to HEOL/HEOR. Note that the HEOL/HEOR in the second cohorts are also attached to the left and right mastoids during the experiment. After pre-processing, the order of electrodes in the first cohort was adjusted to be consistent with the second cohort.

**Table S4. Averaged emotional ratings of the video clips**

| **Video index** | **Joy** | **Tenderness** | **Inspiration** | **Amusement** | **Anger** | **Disgust** | **Fear** | **Sadness** | **Arousal** | **Valence** | **Familiarity** | **Liking** |
| --- | --- | --- | --- | --- | --- | --- | --- | --- | --- | --- | --- | --- |
| 1 | 0.71 | 0.51 | 1.02 | 0.67 | 4.34 | 2.29 | 1.52 | 2.95 | 4.39 | 2.00 | 1.86 | 1.68 |
| 2 | 0.45 | 0.47 | 0.60 | 0.38 | 4.99 | 3.55 | 2.17 | 4.49 | 4.55 | 1.51 | 2.60 | 1.41 |
| 3 | 0.50 | 0.35 | 0.78 | 0.63 | 5.40 | 2.77 | 2.75 | 5.25 | 5.07 | 1.60 | 3.09 | 1.30 |
| 4 | 0.92 | 0.56 | 0.55 | 1.61 | 0.98 | 5.53 | 2.24 | 1.38 | 4.41 | 1.64 | 0.92 | 0.91 |
| 5 | 0.69 | 0.83 | 0.73 | 0.61 | 0.84 | 4.25 | 3.47 | 1.48 | 3.87 | 1.71 | 1.42 | 1.43 |
| 6 | 0.60 | 0.43 | 0.49 | 0.65 | 1.04 | 5.21 | 3.36 | 1.53 | 4.15 | 1.57 | 1.10 | 1.13 |
| 7 | 0.60 | 0.85 | 0.64 | 0.59 | 1.64 | 1.27 | 4.52 | 2.06 | 4.23 | 1.65 | 1.73 | 1.82 |
| 8 | 0.57 | 0.48 | 0.55 | 0.48 | 1.15 | 2.33 | 4.66 | 2.12 | 4.55 | 1.48 | 2.45 | 1.57 |
| 9 | 0.74 | 0.53 | 0.59 | 1.00 | 1.75 | 3.72 | 4.09 | 2.24 | 4.22 | 1.57 | 1.34 | 1.55 |
| 10 | 0.64 | 1.22 | 0.49 | 0.44 | 1.68 | 1.44 | 2.10 | 4.49 | 3.47 | 1.57 | 1.11 | 1.67 |
| 11 | 0.46 | 2.15 | 0.62 | 0.42 | 0.58 | 0.45 | 0.71 | 4.73 | 3.22 | 1.96 | 1.67 | 1.83 |
| 12 | 0.69 | 2.06 | 1.11 | 0.42 | 1.47 | 0.97 | 1.4 | 4.29 | 3.13 | 2.05 | 1.41 | 2.37 |
| 13 | 1.55 | 1.33 | 1.04 | 0.76 | 0.44 | 0.42 | 0.46 | 0.72 | 1.34 | 3.05 | 1.11 | 2.50 |
| 14 | 1.55 | 1.71 | 1.15 | 0.75 | 0.47 | 0.47 | 0.50 | 0.87 | 1.28 | 3.24 | 1.14 | 2.83 |
| 15 | 2.05 | 1.98 | 1.34 | 0.72 | 0.48 | 0.43 | 0.42 | 1.02 | 1.69 | 3.33 | 1.33 | 3.29 |
| 16 | 1.40 | 1.36 | 0.98 | 0.61 | 0.56 | 0.47 | 0.49 | 0.80 | 1.33 | 3.09 | 1.32 | 2.55 |
| 17 | 4.03 | 1.99 | 1.74 | 4.84 | 0.61 | 0.44 | 0.45 | 0.89 | 3.48 | 4.04 | 3.85 | 4.54 |
| 18 | 4.37 | 2.33 | 2.04 | 4.77 | 0.74 | 0.62 | 0.57 | 0.68 | 3.84 | 4.22 | 4.04 | 4.93 |
| 19 | 4.50 | 2.29 | 1.99 | 5.05 | 0.71 | 0.54 | 0.41 | 0.41 | 3.72 | 4.42 | 4.22 | 4.73 |
| 20 | 4.20 | 3.97 | 5.31 | 2.88 | 0.51 | 0.46 | 0.37 | 0.42 | 3.99 | 5.23 | 4.18 | 5.36 |
| 21 | 3.57 | 3.54 | 5.18 | 0.91 | 0.51 | 0.56 | 0.47 | 1.11 | 3.94 | 4.93 | 3.31 | 4.55 |
| 22 | 3.11 | 1.88 | 4.15 | 0.70 | 0.59 | 1.51 | 1.06 | 1.07 | 3.90 | 4.21 | 3.32 | 3.90 |
| 23 | 4.50 | 4.26 | 2.47 | 2.56 | 0.53 | 0.42 | 0.50 | 0.41 | 3.42 | 4.39 | 3.51 | 4.35 |
| 24 | 4.42 | 3.28 | 2.69 | 1.83 | 0.48 | 0.41 | 0.41 | 0.41 | 3.56 | 4.43 | 2.24 | 4.69 |
| 25 | 3.78 | 2.87 | 2.79 | 1.79 | 0.58 | 0.53 | 0.53 | 0.46 | 3.27 | 4.30 | 4.43 | 4.87 |
| 26 | 3.71 | 5.37 | 3.64 | 1.34 | 0.45 | 0.41 | 0.40 | 1.25 | 3.62 | 4.42 | 3.61 | 4.71 |
| 27 | 3.97 | 4.52 | 3.02 | 1.14 | 0.6 | 0.49 | 0.47 | 0.74 | 3.34 | 4.25 | 1.95 | 4.08 |
| 28 | 4.07 | 5.06 | 2.94 | 1.28 | 0.49 | 0.49 | 0.39 | 0.57 | 3.27 | 4.62 | 1.76 | 4.17 |
